# Stability of HIV-1 Nucleic Acids in Dried Blood Spot Samples for HIV-1 Drug Resistance Genotyping

**DOI:** 10.1371/journal.pone.0131541

**Published:** 2015-07-06

**Authors:** Susan C. Aitken, Carole L. Wallis, Wendy Stevens, Tobias Rinke de Wit, Rob Schuurman

**Affiliations:** 1 Department of Medical Microbiology, University Medical Centre Utrecht, Utrecht, The Netherlands; 2 Lancet Laboratories, Johannesburg, South Africa; 3 Department of Molecular Medicine and Hematology, University of the Witwatersrand, Johannesburg, South Africa; 4 National Health Laboratory Services, Johannesburg, South Africa; 5 PharmAccess International, Amsterdam, The Netherlands; 6 Global Health Department, Amsterdam Institute for Global Health and Development (AIGHD), Academic Medical Center, Amsterdam, the Netherlands; Institute of Infection and Global Health, UNITED KINGDOM

## Abstract

Dried blood spots (DBS) are an easy to collect sample-type that can stabilize biological material at ambient temperature for transport and storage, making them ideal for use in resource-limited settings (RLS). We investigated the effect of storage temperature and duration on ability to detect mixed HIV-1 viral RNA populations, and subsequently viral RNA populations in a background of proviral DNA. Part one of the study used DBS samples of whole blood spiked with specific quantities of HIV-1 subtype-B and -C RNA to study mixed virus population detection. Part two used DBS comprising of HIV-1 subtype-B proviral DNA containing U1 cells combined with HIV-1 subtype-C RNA to mimic HIV-1 infected clinical samples as a model system to study the relative stability of HIV-1 RNA and DNA in DBS. Prepared DBS were stored at -20°C and +30°C for periods of one day, one, two, and four weeks. Samples were genotyped to determine changes in the detection of mixtures in the sample over time. From two weeks onwards, storage at +30°C resulted in gradual, time-related reduction in the detection of mixed virus population at log_10_ VL 4.0 but not at log_10_ 5.0. Proviral DNA and viral RNA were both stable for at least 52 weeks when stored at -20°C, compared to progressive RNA decay over time at +30°C. DBS storage conditions and duration had a significant effect on HIV-1 RNA amplification. Our results demonstrate that DBS storage at ambient temperature (+30°C) should not exceed two weeks, with long-term storage at -20°C or lower.

## Introduction

The use of dried blood spot samples (DBS) is becoming increasingly popular for HIV-1 drug resistance genotyping and viral load (VL) monitoring in resource limited settings (RLS). Various studies have been conducted to determine the ability to recover and amplify viral nucleic acid (NA) from DBS samples and the consequent use in various molecular diagnostic applications [1–4]. One important issue is stability of viral RNA in DBS collected, transported, and stored under field conditions in RLS. Findings on this topic are not consistent, most probably due to variations in experimental design and conditions. Results reported demonstrated that samples can be stored at temperatures ranging from -70°C to +37°C for periods of two weeks up to 12 months for VL testing [5–10], and from three months up to six years for genotyping [3,11–13]. Although these reports proved the application of DBS for HIV-1 nucleic acid recovery and amplification, the proportional effect of nucleic acid degradation on the ability to detect mixed virus populations and viral RNA versus proviral DNA at various storage conditions was not always studied.

DBS samples are prepared from whole blood, which comprises a plasma component, harbouring circulating HIV-1 particles containing viral RNA, and a cellular component, including human lymphocytes containing HIV-1 proviral DNA. Genetic diversity between HIV-1 RNA in the virus particles present in the plasma and the HIV-1 proviral DNA in infected cells has been extensively documented [11,14–21]. In addition there is genetic diversity within the viral populations in plasma, due to the high turn-over of virus production and error-prone reverse transcription process that are characteristic of HIV-1 replication [22–24]. This genetic diversity is reflected in viral populations harbouring HIV-1 drug resistance (HIVDR) mutations [25,26]. HIVDR can limit the antiretroviral treatment options for patients, in particular in situations where treatment response is not actively monitored, which frequently is the case in RLS [27]. Early detection of HIVDR, including detection of mutations, is important to maintain future treatment options.

HIVDR genotyping results that are derived from DBS should take into account the potential contribution of proviral DNA of archived viruses to HIVDR profiles. Previous studies comparing genotyping results of DBS and plasma samples prepared from the same blood do not always show consistency; whilst some found comparable results [11,15–17,21,28], others reported noticeable differences [14,18–20]. As DNA is more stable than RNA in DBS, viral RNA in the plasma component may be degraded faster than proviral DNA under suboptimal DBS storage. Consequently, DBS-based HIV-1 genotyping results might reflect disproportional contributions of proviral DNA to the overall determination of HIVDR.

In the current study we investigated the effect of DBS storage temperature and duration on HIV-1 sample nucleic acid integrity. We specifically investigated how degradation of HIV-1 RNA could affect the detection of (emerging) mutations in mixed virus populations; and further how genotypic profiles are influenced by degradation of HIV-1 RNA in the presence of proviral DNA.

## Materials and Methods

### Sample Material: Viruses, Cell Lines, and Whole Blood

Cultured HIV-1 subtypes-B (strain BK132; NCBI accession number AY173951) and C (strain ZB18; NCBI accession number AB4856) from the BBI subtype panel (BBI Biotech Research Laboratories Inc., USA) were used to represent circulating virus. U1 cells [29], each containing one copy of subtype-B proviral DNA, were used to represent latently infected monocytes.

EDTA whole blood (WB) was obtained from the Mini Donor Service (Mini Donor Dienst, MDD) at the University Medical Center Utrecht (UMCU), The Netherlands. The MDD has received positive approval from the Medical Ethics Committee of the UMCU (Medisch Ethische Toetscommissie, METC; www.umcutrecht.nl/metc) for the protocol number 07-125/C (S1 File). The METC concluded that the MDD protocol is not considered medical research but rather falls under the classification of having to abide by the regulations of Good Clinical Practice to maintain confidentiality. Volunteers of the MDD sign an informed consent document prior to partaking in the MDD (S2 File). All MDD participant information is kept confidential and only MDD personal have access to their particulars. WB that is obtained from the MDD is confirmed at being HIV-1 negative by the MDD service.

### Sample Construction

#### Part I

To study the detection of HIV-1 virus harbouring (emerging) drug resistance in a mixed virus population in DBS samples, dilutions of well characterized HIV-1 subtype-B and C viruses were prepared to represent VL of log_10_ 5.0 (high), 4.0 (medium), and 3.0 (low) copies/ml. Viral loads of these dilutions were verified using the HIV-1 COBAS Ampliprep/ COBAS Taqman assay version 2 (Roche, Penzburg, Germany) in duplicate on three separate days to ensure accuracy. Different proportions of HIV-1 subtype-B:C ratios representing 100:0, 90:10, 75:25, 50:50, 25:75, 10:90 and 0:100 percent at high, medium and low VL were prepared using the virus dilutions, which were subsequently used to spike HIV-1 negative WB.

#### Part II

To study the impact of proviral DNA detection in the presence of viral RNA, WB was spiked with U1 cells at a final concentration of 5.00E+04 U1 cells/ml, representing approximately log_10_ 4.7 proviral DNA/ml. HIV-1 subtype-C was added to aliquots of U1-spiked WB to prepare samples containing log_10_ 0.0, 3.7, 4.0, 4.7, 5.4, and 6.0 viral RNA copies/ml. In addition, three RNA controls were prepared in WB containing no U1 cells, representing log_10_ 3.7, 4.7, and 5.4 viral RNA copies/ml.

### Dried Blood Spot Preparation and Storage

Spiked WB was spotted on Whatman 903 filter cards (GE Healthcare, USA). Each card contained five spiked WB spots of 50μl each. After spotting, cards were air dried for three hours in drying racks in a laminar flow hood. Dried cards were placed in zip-lock bags with Minipax indicator desiccant (Sigma-Aldrich, Germany) and stored for time periods of one day (baseline), one, two, and four weeks at either +30°C or -20°C. For part II, the proviral DNA versus viral RNA long-term stability study, cards were additionally stored for 12 and 54 weeks. Additionally in part II we further investigated the effect of short term storage for one day, one, two and four weeks at elevated room temperature followed by two weeks storage at -20°C on RNA and DNA stability.

### Nucleic Acid Isolation

For isolation, two DBS containing 50μl spiked WB each were excised and added to 2ml NucliSENS lysis buffer (BioMerieux, The Netherlands). Samples were incubated for 30 minutes at room temperature with gentle rotation. Filter papers were removed and isolation proceeded using the NucliSENS magnetic extraction reagents in combination with the NucliSENS miniMAG (BioMerieux) according to the manufacturer’s instructions. Samples were eluted in 30ul elution buffer and used immediately for combined cDNA synthesis and amplification. The remaining sample was stored at -20°C. Nucleic acid (NA) isolation and amplification was performed in duplicate for each sample (i.e. two isolations of two spots each, both of which were subsequently genotyped).

### RNA and DNA Genotyping

Isolated NA were amplified and genotyped using an assay targeting a region of the reverse transcriptase (RT) gene from amino acid 41–238 [30], which has a level of detection of log_10_ 3.7 RNA copies/ml. Briefly, the assay amplifies a 560 nucleotide fragment in a single RT-PCR, which is subsequently sequenced using a single forward and a single reverse primer. Sequencing was performed using the BigDye Terminator v3.1 cycle sequencing kit (Life Technologies, USA) and analyzed on an ABI 3730 (Life Technologies).

### Data Analysis

Generated sequences were analyzed and assembled using SeqScape version 2.6 (Life Technologies). The HIV-1 subtype-B virus sequence was used as the reference template for alignment. A total of 45 nucleotide positions were identified at which the subtype-B sequence differs from the subtype-C sequence. These positions were used to determine which subtype, and therefore which virus in a mixed population or NA type, had been detected. Sequences for each sample were manually checked at each of these positions. Based on the results, samples were classified as either pure subtype-B, a mixture of both subtypes-B and C, or pure subtype-C.

## Results

### Part I: Impact of storage temperature and duration on mixed virus populations

Over the periods examined, one day, one week, two and four weeks, nucleic acid amplification was more successful for samples stored at -20°C as compared to +30°C. Amplification of all log_10_ 5.0 samples stored at -20°C (n = 14) and +30°C (n = 14) for one day, one and two weeks was 100% successful, whilst a slight decrease to 86% (12/14) was observed after four weeks storage, but only for samples stored at +30°C. Detection of mixed genotypes in log_10_ 5.0 samples stored at -20°C and +30°C were comparable for all time points (Table 1), indicating a full sensitivity for the detection of viruses in a mixed population.

**Table 1:** Detection of mixed viral populations in dried blood spot samples with two different viral loads, stored at -20°C or +30°C for four different time periods.

			Time Point							
Log VL	Subtype		-20°C				+30°C			
	B	C	1 Day	1 Week	2 Weeks	4 Weeks	1 Day	1 Week	2 Weeks	4 Weeks
**5.0**	**90**	**10**	*Mixed* ^*a*^	*Mixed*	*Mixed*	*Mixed*	*Mixed*	*Mixed*	*Mixed*	*Mixed*
	**75**	**25**	*Mixed*	*Mixed*	*Mixed*	*Mixed*	*Mixed*	*Mixed*	**Pure**	*Mixed*
	**50**	**50**	*Mixed*	*Mixed*	*Mixed*	*Mixed*	*Mixed*	*Mixed*	*Mixed*	*Mixed*
	**25**	**75**	*Mixed*	*Mixed*	*Mixed*	*Mixed*	**Pure**	*Mixed*	*Mixed*	*Mixed*
	**10**	**90**	*Mixed*	*Mixed*	*Mixed*	*Mixed*	*Mixed*	*Mixed*	*Mixed*	**Pure**
**4.0**	**90**	**10**	**Pure** ^**b**^	*Mixed*	**Pure**	*Mixed*	*Mixed*	*Mixed*	**Pure**	**Pure**
	**75**	**25**	*Mixed*	**Pure**	*Mixed*	*Mixed*	*Mixed*	**Pure**	*Mixed*	**Pure**
	**50**	**50**	**Pure**	*Mixed*	*Mixed*	*Mixed*	*Mixed*	**Pure**	*Mixed*	*Mixed*
	**25**	**75**	*Mixed*	*Mixed*	*Mixed*	*Mixed*	*Mixed*	*Mixed*	**Pure**	**Pure**
	**10**	**90**	*Mixed*	**Pure**	*Mixed*	**Pure**	*Mixed*	**Pure**	**Pure**	-^c^

^a^ Mixed: Both subtypes B and C detected.

^b^ Pure: Only one subtype, either B or C, detected.

^c^-: Samples failed to amplify, no genotypic data available.

Amplification of log_10_ 4.0 samples stored at -20°C and +30°C remained comparable for the one day, one and two week time points. At the four week time point, amplification of samples stored at +30°C was slightly decreased to 71% (10/14), compared to 100% (14/14) for samples stored at -20°C. Detection of mixed genotypes in log_10_ 4.0 samples stored at -20°C and +30°C reflected the amplification results, with loss of detection of mixtures only at +30°C after four weeks of storage (Table 1).

For log_10_ 3.0 samples stored at -20°C, amplification success was comparable over the first three time points, 57% (8/14), 43% (6/14) and 43% (6/14), respectively. When stored at +30°C, log_10_ 3.0 samples decreased in amplification success from 36% (5/14) after one day and one week of storage to 29% (4/14) after two weeks storage, and further down to 14% (2/14) after four weeks of storage. Genotyping results for log_10_ 3.0 samples stored at -20°C and +30°C for all time points indicated that only single species were amplified (32/80), either subtype-B or C. Mixtures of B/C were detected in only eight of the 80 samples (data not shown).

### Part II: Impact of storage temperature and duration on viral RNA versus proviral DNA

Amplification of all DNA-RNA samples was achieved at all time points for both storage at -20°C and +30°C. A mixture of RNA and DNA was detected at concentrations where the DNA ration was between 20–80%. Data indicated that both proviral DNA and viral RNA were stable for at least one year when stored at -20°C, whereas when stored at +30°C there was progressive loss of RNA detection at each time-point (Table 2). DBS samples stored at +30°C resulted in an increased preferential detection of proviral DNA versus viral RNA over time compared to the baseline profile, with proviral DNA detection being most prominent at lower RNA concentrations. Storage longer than one week at +30°C resulted in a progressive decline of RNA detection. After one year of storage at +30°C, only proviral DNA was amplified and viral RNA was no longer detectable at any of the concentrations tested (Table 2).

**Table 2:** Nucleic acid stability of viral RNA and proviral DNA in dried blood spot samples stored at -20°C or +30°C for six different time points.

		Time Point											
Log VL		-20°C						+30°C					
DNA	RNA	1 day	1 week	2 weeks	4 weeks	12 weeks	52 weeks	1 day	1 week	2 weeks	4 weeks	12 weeks	52 weeks
4.7	6.0	RNA^a^	RNA	RNA	RNA	RNA	RNA	RNA	RNA	RNA	Both	Both	***DNA***
	5.4	Both	RNA	RNA	RNA	RNA	RNA	Both	Both	Both	Both	Both	***DNA***
	4.7	Both	Both	Both	Both	Both	Both	Both	Both	***DNA***	***DNA***	***DNA***	***DNA***
	4.0	Both^b^	Both	Both	Both	Both	Both	***DNA***	***DNA***	***DNA***	***DNA***	***DNA***	***DNA***
	3.7	***DNA*** ^c^	***DNA***	***DNA***	***DNA***	***DNA***	***DNA***	***DNA***	***DNA***	***DNA***	***DNA***	***DNA***	***DNA***

^a^RNA: RNA detected, DNA not detected.

^b^Both: DNA and RNA detected

^c^DNA detected, RNA not detected; Values where RNA was not detected are emphasised in bold

To mimic field application of DBS collection, an experiment was performed in which short term storage at (elevated) room temperature was followed by storage at -20°C. Amplification of all DNA-RNA samples following sample storage for one day (baseline), one and two weeks at +30°C, followed by two weeks at -20°C, was successful for all samples. After four weeks storage at +30°C, followed by two weeks at -20°C, none of the log_10_ 3.0 RNA-only samples that were stored at +30°C were amplified, whereas those stored at -20°C for the six weeks could all be successfully amplified. Genotyping of samples stored at +30°C for two weeks, followed by -20°C for another two weeks, demonstrated that the results were comparable to baseline results. After four week storage at +30°C, followed by two weeks at -20°C, the loss of RNA detection was notable at the highest VL concentration of DNA-RNA samples (Table 3).

**Table 3:** Nucleic acid stability of viral RNA and proviral DNA in dried blood spot samples stored at +30°C for four different time points, each followed by two weeks storage at -20°C.

Log VL		Time Point			
DNA	RNA	1 day	1 week	2 weeks	4 weeks
	3.7	***DNA*** ^***a***^	***DNA***	***DNA***	***DNA***
4.7	4.0	***DNA***	***DNA***	***DNA***	***DNA***
	4.7	Both^b^	Both	Both	Both
	5.4	Both	Both	Both	Both
	6.0	RNA^c^	RNA	RNA	Both

^a^DNA detected, RNA not detected; Values where RNA was not detected are emphasised in bold

^b^Both: DNA and RNA detected

^c^RNA: RNA detected, DNA not detected.

## Discussion

We have taken a unique approach to study the effects of storage duration and temperature on the differential integrity of HIV-1 nucleic acids in DBS samples by using mixtures of two different HIV-1 subtypes as a proxy for mixed virus populations. This approach gives a precise, easily interpretable result, and avoids sample manipulation, such as DNAse treatments. The results of our study demonstrate that long term storage of DBS samples at +30°C has a negative impact on genotyping of HIV-1 RNA, especially at lower VL, and that DBS sample storage at -20°C is ideal to maintain sample integrity. High ambient temperatures (+30°C) lead to progressive degradation of viral RNA, resulting in selective amplification of remaining RNA, which in turn leads to the loss of detection of different viruses present in a mixed population. Furthermore, we also demonstrated that loss of viral RNA subsequently results in preferential amplification of proviral DNA. Our results indicate that maintaining the quality of the DBS sample for VL and HIVDR determination on the one hand and using simple sample collection and shipment procedures on the other can be achieved if RT storage of DBS does not exceed two weeks, with subsequent long term storage at -20°C or below.

The method we have used for sample analysis utilizes population-based genotyping, which has previously been shown to be capable of detecting minority viral populations when they constitute greater than 20% of the population [31–34]. Our current results indicate that detection of minority viral populations can be at the level of 10% in samples with a log_10_ VL greater than 4.0. We postulate that the sensitivity of population-based HIVDR assays is influenced more by the number of copies of the drug resistant viruses within the population than by their percentage within the population. This conclusion is further supported by the failure to detect both subtypes in our samples with log_10_ VL of 3.0. Recent work by Rottinghaus *et al*. has also suggested that mutation detection and nucleic acid integrity may also be influenced by the type of filter paper used for DBS collection[35]. This may be a result of the efficiency of re-suspending the sample from the filter paper, with poor re-suspension resulting in a decreased copy number of minority virus populations which subsequently may be missed by population-based genotyping.

Whilst previous studies have shown comparable results for genotyping of viral RNA and proviral DNA [11,15–17,21], others gave more controversial results [14,18–20]. In the latter case, sometimes more mutations were detected in the proviral DNA as compared to viral RNA [19,20], and sometimes vice versa [15,36]. It has previously been suggested that a combined DNA-RNA total genotypic profile would provide a complete overview of current and potential future drug resistance [15]. This suggestion is contradicted by other research indicating that the majority of proviral DNA found in peripheral blood mononuclear cells (PBMCs) is defective and will potentially never progress to integration [22,37,38]. In addition, it has been shown that virus producing cells are often cleared quickly by the immune system, and that quiescent infected T-cells, which represent future potential emergence of resistance, make up a very small proportion of PBMCs, as little as <0.01% [37,39,40]. Taken together, these results indicate that HIVDR profiles generated from proviral DNA may not always be conclusive and do not necessarily provide information about current or future circulating viral populations, highlighting the importance of maintaining viral RNA integrity in DBS samples in order to prevent predominant genotyping of proviral DNA.

The above mentioned influence of viral RNA degradation and the impact of proviral DNA amplification on HIVDR determination could also play a role in VL testing using DBS. Amplification success with regard to RNA degradation will be dependent on the size of the target amplicon [9]. Typical real-time PCR-based VL assays target a smaller region of the genome [41,42] compared to HIVDR assays [43,44]. Therefore such VL assays may be less sensitive to result in underestimated or false-negative VL results when applied on DBS.

## Conclusions

DBS storage conditions and duration have a significant effect on the representation of the type of nucleic acid being amplified and as such could affect the profile of HIV-1 drug resistance patterns detected. In samples with a low VL, loss of sample integrity will result in early loss of viruses present as minority species within a mixed viral population, and simultaneous risk of predominant amplification of archived proviral DNA. Our results indicate that DBS storage at ambient temperature (+30°C) should not exceed two weeks and long term storage should be at -20°C or lower.

## Supporting Information

S1 File Medical Ethics (METC) Approval Letter (Dutch only). This letter contains the approval from the Medical Ethics Commitee (Medisch Ethische Toetscommissie, METC; www.umcutrecht.nl/metc) for the protocol number 07-125/C. This protocol covers the service known as the Mini Donor Dienst (MDD), which is a voluntary service through which people can donate blood for use in research.
(PDF)

S2 File Informed consent form for the Mini Donor Diesnt (MDD) (Dutch only). This document is the informed consent form that is used to obtain consent from people who wish to donate blood for the MDD.
(PDF)
