# Supplementary material for: Stability of HIV-1 Nucleic Acids in Dried Blood Spot Samples for HIV-1 Drug Resistance Genotyping
Source: PLoS One. 2015 Jul 6;10(7):e0131541. doi: 10.1371/journal.pone.0131541 (PMC4493047; doi:10.1371/journal.pone.0131541)
Supplement: S1 File — This letter contains the approval from the Medical Ethics Commitee (Medisch Ethische Toetscommissie, METC; www.umcutrecht.nl/metc) for the protocol number 07-125/C. This protocol covers the service known as the Mini Donor Dienst (MDD), which is a voluntary service through which people can donate blood for use in research. (PDF) [file pone.0131541.s001.pdf]

Universitair Medisch Centrum
Utrecht
Medisch Ethische Toetsingscommissie

Divisie Laboratoria en Apotheek
Afdeling Hematologie
t.a.v. Prof.dr. J.W.N. Akkerman
Huispost : G03.647

Mw. M.A.C. van Groenestijn
Telefoon 088-7556376 (ma t/m do 09.30-12.00uur)
Heidelberglaan 100
Postbus 85500
3508 GA Utrecht
Huispost D 01.343
E-mail metc@umcutrecht.nl
Info www.umcutrecht.nl/metc

Datum
18 december 2007

Onderwerp
METC-protocolnummer 07-125/C
Positief Advies niet-WMO onderzoek

Ons kenmerk
AvG/vb/07/23245

Uw kenmerk

Geachte heer Akkerman,

De Medisch Ethische Toetsingscommissie (METC), erkend op 11 november 1999 ex artikel 16 van de WMO heeft zich in de vergadering van 11 december 2007 beraden over het onderzoeksvoorstel nummer 07/125, getiteld **"Mini Donor Dienst"**, ingediend door Prof.dr. J.W.N. Akkerman , met als verrichter UMC Utrecht.

De METC van het UMC Utrecht heeft zich op het standpunt gesteld dat het ingediende onderzoeksvoorstel niet in de zin van de WMO getoetst hoeft te worden. Hierbij heeft de commissie overwogen dat er geen sprake is van het onderwerpen van proefpersonen aan handelingen of het opleggen van een gedragswijze zoals bedoeld in de definitie van medisch-wetenschappelijk onderzoek in de WMO (art. 1b).

De commissie heeft het onderzoeksprotocol dan ook alleen getoetst in het kader van de zorgvuldigheid ter verkrijging van een verklaring van geen bezwaar. Het toetsingskader is derhalve niet de WMO, maar de code "Goed Gebruik", de code "Goed Gedrag" alsmede de Wet bescherming persoonsgegevens (Wbp). De code "Goed Gebruik" stelt gedragsregels vast voor wetenschappelijke onderzoekers die gebruik willen maken van lichaamsmateriaal dat in eerste instantie voor een ander doel is afgenomen. De code "Goed Gedrag" vormt een uitwerking van met name het bepaalde in de Wet op de geneeskundige behandelingsovereenkomst over het gebruik van patiëntengegevens in wetenschappelijk onderzoek. Dit betekent onder meer dat voor het gebruik van medische gegevens van patiënten ten behoeve van wetenschappelijk onderzoek in beginsel toestemming van de patiënten vereist is (art. 458 WGBO). De zorgvuldigheidstoets heeft geleid tot het volgende.

De commissie is van oordeel dat het een zorgvuldig opgezet onderzoek betreft dat voldoet aan het bovengenoemde toetsingskader. De commissie heeft dan ook geen bezwaar tegen de uitvoering van het onderzoek, zoals omschreven in het protocol d.d. 05 november 2003 en de patiënteninformatie en het informed consent formulier d.d. 06 november 2007. Wellicht ten overvloede merkt de commissie op, dat een toetsing in het kader van de zorgvuldigheid niet gezien mag worden als een oordeel in de zin van de WMO.

9401168

Ingevolge de "Code Goed Gedrag", de "Code Goed Gebruik" en de Wet bescherming **persoonsgegevens** (Wbp) dient zoveel mogelijk gebruik te worden gemaakt van anonieme gegevens. Indien dat niet mogelijk is, dient zoveel mogelijk gebruik te worden gemaakt van gecodeerde persoonsgegevens. Als ook dat niet mogelijk is, is het toegestaan gebruik te maken van direct identificerende persoonsgegevens. Onder 'zoveel mogelijk' wordt verstaan zoveel als verantwoord is, gelet op de aard en het doel van het onderzoek en de juiste uitvoering daarvan. In het onderzoeksprotocol dient te worden aangegeven, voor zover geen anonieme gegevens worden gebruikt, de reden waarom het gebruik van gecodeerde of direct herleidbare persoonsgegevens noodzakelijk is. Tevens dient in het onderzoeksprotocol te worden aangegeven wie toegang hebben tot de sleutel van de code dan wel wie toegang hebben tot de direct herleidbare persoonsgegevens. De tekst van beide codes kunt u vinden op de website van de Federatie van Medisch Wetenschappelijke Verenigingen: www.fmwv.nl.

Iedere wijziging van de onderzoeksopzet, hoe ogenschijnlijk gering ook, dient u opnieuw voor toestemming aan de METC voor te leggen. Mocht het onderzoek in de toekomst op een zodanige wijze gewijzigd worden dat het alsnog onder de reikwijdte van de WMO zou vallen, dan dient u het onderzoek opnieuw voor beoordeling aan de METC voor te leggen.

Met vriendelijke groeten,
namens de METC,

M.A.C. van Groenestijn
Secretaris

Een lijst van de samenstelling van de commissie en het reglement van de commissie zijn op te vragen bij het secretariaat van de METC.

9401168

Universitair Medisch Centrum
Utrecht

Medisch Ethische Toetsingscommissie

Divisie Laboratoria en Apotheek
Afdeling Hematologie
t.a.v. Prof.dr. J.W.N. Akkerman
Huispost : G03.550

Mw. M.A.C. van Groenestijn
Telefoon 088-7556376 (ma t/m do 09.30-12.00uur)
Heidelberglaan 100
Postbus 85500
3508 GA Utrecht
Huispost D 01.343
E-mail metc@umcutrecht.nl
Info www.umcutrecht.nl/metc

CCMO nummer: NL@@@

Datum
02 maart 2010

Ons kenmerk
AvG/ad/10/05641

Onderwerp
METC-protocolnummer 07-125/C
Positief advies amendement

Uw kenmerk
...

Geachte heer Akkerman,

De Medisch Ethische Toetsingscommissie (METC), erkend op 11 november 1999 ex artikel 16 van de WMO heeft zich in de vergadering van 19 januari 2010 beraden over het amendement op het onderzoeksvoorstel nummer 07/125, getiteld **"Mini Donor Dienst"**, ingediend door Prof.dr. J.W.N. Akkerman te Utrecht, met als verrichter UMC Utrecht te Utrecht.

De commissie heeft in haar vergadering de beschikking gehad over de volgende documenten:

1. Amendement, d.d. 24 december 2009, ontvangen 28 december 2009, betreffende gewijzigde procedurebeschrijvingen vanwege een veranderde organisatie van de bloedafnames
2. Formulier Mini Donor Dienst (MDD) betreft Laboratorium Klinische Chemie en Haematologie (LKCH) van het UMC Utrecht, versie 004, d.d. 07 december 2009, ontvangen 28 december 2009
3. Formulier Mini Donor Dienst (MDD) betreft Department of Clinical Chemistry and Haematology (DCCH) van het UMC Utrecht, versie 004, d.d. 07 december 2009, ontvangen 28 december 2009

De commissie verbindt aan het amendement een positief advies.

Iedere wijziging van het protocol, hoe ogenschijnlijk gering ook, dient u opnieuw voor toestemming aan de METC voor te leggen.

Met vriendelijke groeten,
mede namens de Raad van Bestuur,

M.A.C. van Groenestijn,
Secretaris

Een lijst van de samenstelling van de commissie en het reglement van de commissie zijn op te vragen bij het secretariaat van de METC.

9401168
