# Supplementary material for: Stability of HIV-1 Nucleic Acids in Dried Blood Spot Samples for HIV-1 Drug Resistance Genotyping
Source: PLoS One. 2015 Jul 6;10(7):e0131541. doi: 10.1371/journal.pone.0131541 (PMC4493047; doi:10.1371/journal.pone.0131541)
Supplement: S2 File — This document is the informed consent form that is used to obtain consent from people who wish to donate blood for the MDD. (PDF) [file pone.0131541.s002.pdf]

Aanmeldingsformulier MDD / Informed Consent

Mini Donor Dienst
Laboratorium Klinische Chemie en Haematologie
Huispostnr. G03.550
Postbus 85.500
3508 GA UTRECHT

Tel.: 088 – 755.76.13
Fax: 088 – 755.54.18
E-mail: mddumc@umcutrecht.nl

Bloedafgifte ten behoeve van wetenschappelijk onderzoek in het UMC Utrecht

Ik verklaar hierbij bereid te zijn tot deelname aan de Mini Donor Dienst van het Universitair Medisch Centrum Utrecht, voor vrijwillige donaties van bloed voor wetenschappelijke doeleinden, niet vaker dan vier keer per jaar en niet meer dan 100 ml per afname.

Ik verklaar gezond te zijn, 18 jaar of ouder en wilsbekwaam.

Ik verklaar **wel/geen*** donor te zijn bij de bloedtransfusiedienst Sanquin Bloedbank Noord-West veranderingen in deze situatie zullen worden gemeld aan de organisatie van Mini Donor Dienst.

Ik geef toestemming bij elke donatie t.b.v. de Mini Donor Dienst voor het afnemen van 6 ml bloed voor de bepaling van HbsAg (anti-HBV), anti-HCV en anti-HIV. Bij een eventuele positieve uitslag van één of meerdere testen zal ik hiervan op de hoogte worden gesteld door een arts van de Afdeling Infectieziekten, waar deze testen worden uitgevoerd.

Ik bevestig dat ik het informatieformulier voor de proefpersoon (B3P50) heb gelezen. Ik begrijp de informatie. Ik heb de gelegenheid gehad om aanvullende vragen te stellen. Deze vragen zijn in voldoende mate beantwoord. Ik heb voldoende tijd gehad om over deelname na te denken.

Ik weet dat mijn deelname geheel vrijwillig is en dat ik mijn toestemming op ieder moment kan intrekken zonder dat ik daarvoor een reden hoeft te geven.

Ik geef toestemming om op de hoogte te worden gesteld van onverwachte bevindingen waarvoor medisch behandelen noodzakelijk wordt geacht.

Ik geef toestemming om de gegevens te verwerken voor de doeleinden zoals beschreven in de informatiebrief.

Ik geef toestemming voor het bewaren en gebruiken van gegevens voor eventueel vervolg onderzoek.

Ik geef **wel/geen*** toestemming om lichaamsmateriaal in de toekomst eventueel te gebruiken voor onderzoek met een zelfde onderzoeksdoel.

Ik ben bekend met het feit dat het bloed gecodeerd wordt verwerkt teneinde mijn privacy the waarborgen. Alleen bij positieve besmettingscontroles zal het hoofd Mini Donor Dienst de code opheffen en mijn gegevens vertrouwelijk aanbieden aan de arts van de Afdeling Infectieziekten die mij nader zal informeren.

Ik stem in met mijn deelname aan bovengenoemd onderzoek.

Aanmeldingsformulier MDD / Informed Consent

Roepnaam	:	Bloedgroep (indien bekend)	:
Achternaam	:	Huisarts	:
Adres	:	Afdeling	:
Postcode	:	Huispostnummer (Hp.nr)	:
Plaats	:	Tel. Werk / Zoemernummer	:
Tel. Privé	:	Geboortedatum	:

Handtekening:

Datum: __ / __ / 20__

Ik heb mondelinge en schriftelijke toelichting verstrekt op het onderzoek. Ik verklaar mij bereid nog opkomende vragen over het onderzoek naar vermogen te beantwoorden.

Naam Mini Donor Dienst medewerker:

Handtekening:

Datum: __ / __ / 20__

*** Doorhalen wat niet van toepassing is.**
